# *N*-(3,4-Difluoro­phen­yl)-*N*′-(2,5-di­methoxy­phen­yl)urea

**DOI:** 10.1107/S1600536810032095

**Published:** 2010-08-18

**Authors:** Hyeong Choi, Byung Hee Han, Taewoo Lee, Sung Kwon Kang, Chang Keun Sung

**Affiliations:** a Department of Chemistry, Chungnam National University, Daejeon 305-764, Republic of Korea; b Department of Food Science and Technology, Chungnam National University, Daejeon 305-764, Republic of Korea

## Abstract

In the title compound, C_15_H_14_F_2_N_2_O_3_, the dihedral angle between the benzene rings is 64.5 (1)°. One F atom is disordered over two *meta* positions, with occupancy factors of 0.72 and 0.28. In the crystal, mol­ecules are linked by N—H⋯O hydrogen bonds involving two N—H and one C=O groups of the urea central fragment, leading to a supra­molecular chain along [011].

## Related literature

For general background to the development of potent inhib­itory agents of tyrosinase and melanin formation used as whitening agents, see: Cabanes *et al.* (1994 ▶); Choi *et al.* (2010 ▶); Criton & Le Mellay-Hamon (2008 ▶); Germanas *et al.* (2007 ▶); Dawley & Flurkey (1993 ▶); Ha *et al.* (2007 ▶); Hong *et al.* (2008 ▶); Kwak *et al.* (2010 ▶); Lee *et al.* (2007 ▶); Nerya *et al.* (2003 ▶); Yi *et al.* (2009 ▶, 2010 ▶).
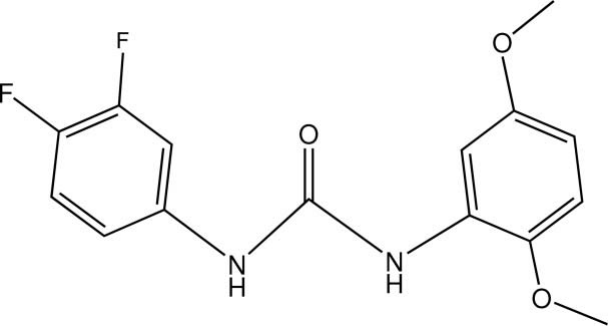


## Experimental

### 

#### Crystal data

C_15_H_14_F_2_N_2_O_3_
*M* *_r_* = 308.28
Monoclinic, 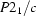

*a* = 13.209 (2) Å
*b* = 12.0887 (18) Å
*c* = 9.0740 (12) Å
β = 104.990 (4)°
*V* = 1399.6 (4) Å^3^
*Z* = 4
Mo *K*α radiation
μ = 0.12 mm^−1^
*T* = 174 K
0.09 × 0.04 × 0.02 mm

#### Data collection

Bruker SMART CCD area-detector diffractometer
10104 measured reflections
2433 independent reflections
1211 reflections with *I* > 2σ(*I*)
*R*_int_ = 0.120

#### Refinement

*R*[*F* ^2^ > 2σ(*F* ^2^)] = 0.061
*wR*(*F* ^2^) = 0.145
*S* = 0.96
2433 reflections
218 parameters
H atoms treated by a mixture of independent and constrained refinement
Δρ_max_ = 0.25 e Å^−3^
Δρ_min_ = −0.26 e Å^−3^

### 

Data collection: *SMART* (Bruker, 2002 ▶); cell refinement: *SAINT* (Bruker, 2002 ▶); data reduction: *SAINT*; program(s) used to solve structure: *SHELXS97* (Sheldrick, 2008 ▶); program(s) used to refine structure: *SHELXL97* (Sheldrick, 2008 ▶); molecular graphics: *ORTEP-3 for Windows* (Farrugia, 1997 ▶); software used to prepare material for publication: *WinGX* (Farrugia, 1999 ▶).

## Supplementary Material

Crystal structure: contains datablocks global, I. DOI: 10.1107/S1600536810032095/bh2300sup1.cif

Structure factors: contains datablocks I. DOI: 10.1107/S1600536810032095/bh2300Isup2.hkl

Additional supplementary materials:  crystallographic information; 3D view; checkCIF report

## Figures and Tables

**Table 1:** Hydrogen-bond geometry (Å, °)

*D*—H⋯*A*	*D*—H	H⋯*A*	*D*⋯*A*	*D*—H⋯*A*
N9—H9⋯O11^i^	0.84 (4)	2.09 (4)	2.907 (4)	163 (4)
N12—H12⋯O11^i^	0.80 (4)	2.30 (4)	3.002 (4)	147 (4)

Symmetry code: (i) 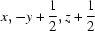.

**Fig. 1.**
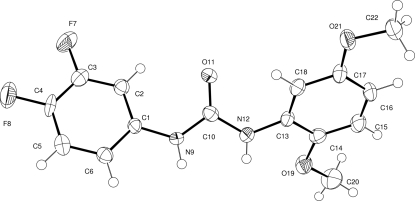
Molecular structure of (l), showing the atom-numbering scheme and 50% probability ellipsoids.

## supplementary crystallographic information

### Comment

Melanin is the pigment responsible for the color of human skin and it is formed through a series of oxidative reaction in the presence of key enzyme tyrosinase (Ha *et al.*, 2007) that converts tyrosine into melanin. It is secreted by melanocyte cells distributed in the basal layer of the dermis. Its role is to protect the skin from ultraviolet (UV) damage by absorbing the ultraviolet sunlight and removing reactive oxygen species. Therefore these inhibitors are target molecules for developing anti-pigmentation agents. Common tyrosinase inhibitors (Dawley & Flurkey, 1993; Nerya *et al.*, 2003) are hydroquinone, ascorbic acid, kojic acid and arbutin (Cabanes *et al.*, 1994). Recently, numerous reports have focused on the inhibition of tyrosinase. They are containing aromatic, methoxy, hydroxyl (Hong *et al.*, 2008; Lee *et al.*, 2007), aldehyde (Yi *et al.*, 2010), amide (Kwak *et al.*, 2010; Choi *et al.*, 2010), thiosemicarbazone (Yi *et al.*, 2009), thiazole (Germanas *et al.*, 2007), thiourea (Criton & Le Mellay-Hamon, 2008) groups in their structures, and act as a specific functional group to make the skin white by inhibiting the production of melanin. However, most of them are not potent enough to put into practical use due to their weak individual activities, poor skin penetration, and low stability of formulations, as well as toxicity or safety concerns. Consequently, there is still need to search and develop novel tyrosinase inhibitors with better activities together with lower side effects. To complement the inadequacy of current whitening agent above mentioned and maximize the effect of inhabitation of melanin creation, we have synthesized the title compound, (I), from the reaction of 3,4-difluoroaniline with 2,5-dimethoxyphenyl isocyanate, under ambient conditions. Herein, the crystal sturucture of (I) is described (Fig. 1).

The 3,4-difluoroaniline group and 2,5-dimethoxyphenyl moiety are essentially planar, with a mean deviations of 0.007 Å and 0.016 Å, respectively, from the corresponding least-squares planes defined by each nine constituent atoms. The dihedral angle between the benzene rings is 64.5 (1) °. The presence of intermolecular N—H···O hydrogen bonds lead to the formation a supramolecular chain along [011].

### Experimental

2,5-Dimethoxyphenyl isocyanate and 3,4-difluoroaniline were purchased from Sigma Chemical Co. Solvents used for synthesis were redistilled before use. All other chemicals and solvents were of analytical grade and used without further purification. The title compound was prepared from the reaction of 3,4-difluoroaniline (0.28 ml, 3 mmol) and 2,5-dimethoxyphenyl isocyanate (0.5 g, 3 mmol) in acetonitrile (6 ml). The mixture was refluxed for 8 h at 353 K, and then treated with water and extracted with methylene chloride (2 × 50 mL). The combined extracts were dried over anhydrous magnesium sulfate. Removal of solvent gave a white solid (90%, m.p. 454 K). Single crystals were obtained by slow evaporation of a methylene chloride solution at room temperature.

### Refinement

The amide H atoms were located in a difference map and refined freely. The remaining H atoms were positioned geometrically and refined using a riding model with C—H = 0.93–0.96 Å, and with *U*_iso_(H) = 1.2*U*_eq_ (C) for aromatic and 1.5*U*_eq_(C) for methyl H atoms. Atom F7 is disordered over two positions and the two split atoms are designated by having the suffix A after the atom number. The final occupancy factors are F7 0.72 and F7A 0.28. The measured diffraction fraction is relatively low of 95.5% due to a tiny single-crystal for data collection. This single-crystal was the largest one we could produce as described in the experimental section.

### Crystal data

**Table:**

C_15_H_14_F_2_N_2_O_3_	*F*(000) = 640
*M**_r_* = 308.28	*D*_x_ = 1.463 Mg m^−^^3^
Monoclinic, *P*2_1_/*c*	Melting point: 454 K
Hall symbol: -P 2ybc	Mo *K*α radiation, λ = 0.71073 Å
*a* = 13.209 (2) Å	Cell parameters from 398 reflections
*b* = 12.0887 (18) Å	θ = 3.0–18.6°
*c* = 9.0740 (12) Å	µ = 0.12 mm^−^^1^
β = 104.990 (4)°	*T* = 174 K
*V* = 1399.6 (4) Å^3^	Needle, colourless
*Z* = 4	0.09 × 0.04 × 0.02 mm

### Data collection

**Table:**

Bruker SMART CCD area-detector diffractometer	*R*_int_ = 0.120
Radiation source: fine-focus sealed tube	θ_max_ = 25.3°, θ_min_ = 1.6°
φ and ω scans	*h* = −15→14
10104 measured reflections	*k* = −12→14
2433 independent reflections	*l* = −4→10
1211 reflections with *I* > 2σ(*I*)	

### Refinement

**Table:**

Refinement on *F*^2^	0 restraints
Least-squares matrix: full	0 constraints
*R*[*F*^2^ > 2σ(*F*^2^)] = 0.061	H atoms treated by a mixture of independent and constrained refinement
*wR*(*F*^2^) = 0.145	*w* = 1/[σ^2^(*F*_o_^2^) + (0.0573*P*)^2^] where *P* = (*F*_o_^2^ + 2*F*_c_^2^)/3
*S* = 0.96	(Δ/σ)_max_ < 0.001
2433 reflections	Δρ_max_ = 0.25 e Å^−^^3^
218 parameters	Δρ_min_ = −0.26 e Å^−^^3^

### Fractional atomic coordinates and isotropic or equivalent isotropic displacement parameters (Å^2^)

**Table:**

	*x*	*y*	*z*	*U*_iso_*/*U*_eq_	Occ. (<1)
C1	0.6480 (3)	0.4327 (3)	0.4129 (4)	0.0243 (9)	
C2	0.5993 (3)	0.4687 (3)	0.2657 (4)	0.0278 (10)	
H2	0.5991	0.4245	0.1818	0.033*	
C3	0.5517 (3)	0.5705 (4)	0.2464 (4)	0.0351 (11)	
H3	0.5189	0.5949	0.1484	0.042*	0.28
C4	0.5517 (3)	0.6365 (3)	0.3683 (5)	0.0354 (11)	
C5	0.5984 (3)	0.6018 (4)	0.5137 (4)	0.0336 (11)	
H5	0.5973	0.6468	0.5964	0.04*	0.72
C6	0.6469 (3)	0.5008 (3)	0.5371 (4)	0.0278 (10)	
H6	0.6791	0.4775	0.6358	0.033*	
F7	0.5055 (3)	0.6079 (3)	0.1088 (3)	0.0597 (11)	0.72
F7A	0.6007 (6)	0.6720 (7)	0.6227 (8)	0.041 (2)	0.28
F8	0.5028 (2)	0.7365 (2)	0.3447 (3)	0.0586 (8)	
N9	0.6969 (2)	0.3289 (3)	0.4443 (3)	0.0274 (9)	
H9	0.706 (3)	0.302 (3)	0.533 (4)	0.038 (12)*	
C10	0.7429 (3)	0.2699 (3)	0.3512 (4)	0.0271 (10)	
O11	0.74653 (19)	0.3012 (2)	0.2231 (2)	0.0301 (7)	
N12	0.7839 (3)	0.1710 (3)	0.4127 (4)	0.0315 (9)	
H12	0.775 (3)	0.151 (3)	0.492 (4)	0.036 (13)*	
C13	0.8518 (3)	0.1019 (3)	0.3556 (4)	0.0259 (10)	
C14	0.8499 (3)	−0.0108 (4)	0.3854 (4)	0.0295 (10)	
C15	0.9184 (3)	−0.0817 (4)	0.3393 (4)	0.0347 (11)	
H15	0.9177	−0.1569	0.3604	0.042*	
C16	0.9883 (3)	−0.0401 (4)	0.2614 (4)	0.0346 (11)	
H16	1.0341	−0.0875	0.2299	0.042*	
C17	0.9893 (3)	0.0715 (4)	0.2313 (4)	0.0317 (10)	
C18	0.9216 (3)	0.1432 (3)	0.2788 (4)	0.0295 (10)	
H18	0.9233	0.2186	0.259	0.035*	
O19	0.7767 (2)	−0.0423 (2)	0.4625 (3)	0.0381 (7)	
C20	0.7657 (4)	−0.1587 (4)	0.4843 (5)	0.0496 (13)	
H20A	0.7476	−0.1951	0.387	0.074*	
H20B	0.7114	−0.1709	0.5353	0.074*	
H20C	0.8307	−0.1879	0.5453	0.074*	
O21	1.0554 (2)	0.1220 (2)	0.1555 (3)	0.0414 (8)	
C22	1.1393 (3)	0.0546 (4)	0.1310 (4)	0.0423 (12)	
H22A	1.18	0.0265	0.2271	0.063*	
H22B	1.1833	0.0984	0.0846	0.063*	
H22C	1.1107	−0.006	0.0651	0.063*	

### Atomic displacement parameters (Å^2^)

**Table:**

	*U*^11^	*U*^22^	*U*^33^	*U*^12^	*U*^13^	*U*^23^
C1	0.029 (2)	0.018 (2)	0.0284 (19)	−0.0011 (19)	0.0118 (16)	0.0004 (16)
C2	0.030 (2)	0.024 (3)	0.0296 (19)	−0.001 (2)	0.0088 (17)	−0.0009 (17)
C3	0.034 (3)	0.034 (3)	0.036 (2)	0.004 (2)	0.0063 (19)	0.007 (2)
C4	0.034 (3)	0.016 (3)	0.055 (3)	0.010 (2)	0.010 (2)	0.004 (2)
C5	0.030 (3)	0.028 (3)	0.045 (2)	0.000 (2)	0.0121 (19)	−0.006 (2)
C6	0.030 (2)	0.027 (3)	0.0278 (19)	−0.003 (2)	0.0082 (16)	−0.0031 (17)
F7	0.077 (3)	0.052 (3)	0.0428 (18)	0.022 (2)	0.0015 (17)	0.0155 (18)
F7A	0.050 (6)	0.031 (5)	0.050 (4)	0.006 (5)	0.025 (4)	−0.016 (4)
F8	0.066 (2)	0.0366 (18)	0.0715 (16)	0.0211 (15)	0.0138 (14)	0.0024 (13)
N9	0.039 (2)	0.025 (2)	0.0191 (16)	0.0072 (18)	0.0103 (14)	0.0042 (15)
C10	0.025 (3)	0.031 (3)	0.0251 (19)	−0.001 (2)	0.0065 (16)	−0.0030 (18)
O11	0.0381 (17)	0.0301 (18)	0.0248 (12)	0.0040 (14)	0.0133 (11)	0.0004 (11)
N12	0.043 (2)	0.025 (2)	0.0300 (18)	0.0083 (18)	0.0158 (16)	0.0063 (16)
C13	0.029 (3)	0.021 (3)	0.0270 (19)	0.004 (2)	0.0059 (17)	0.0016 (17)
C14	0.035 (3)	0.026 (3)	0.0265 (19)	−0.003 (2)	0.0066 (18)	0.0019 (18)
C15	0.046 (3)	0.020 (3)	0.037 (2)	0.003 (2)	0.008 (2)	−0.0021 (18)
C16	0.042 (3)	0.027 (3)	0.036 (2)	0.011 (2)	0.0121 (19)	−0.0048 (19)
C17	0.033 (3)	0.024 (3)	0.039 (2)	0.007 (2)	0.0094 (19)	0.0010 (19)
C18	0.037 (3)	0.018 (3)	0.034 (2)	0.001 (2)	0.0105 (18)	0.0033 (17)
O19	0.0450 (19)	0.0241 (19)	0.0492 (15)	−0.0003 (15)	0.0193 (14)	0.0043 (13)
C20	0.066 (3)	0.028 (3)	0.057 (3)	−0.012 (3)	0.019 (2)	0.003 (2)
O21	0.0436 (19)	0.035 (2)	0.0553 (16)	0.0122 (15)	0.0299 (14)	0.0078 (14)
C22	0.043 (3)	0.041 (3)	0.050 (2)	0.012 (2)	0.024 (2)	0.003 (2)

### Geometric parameters (Å, °)

**Table:**

C1—C2	1.394 (5)	C13—C14	1.390 (5)
C1—C6	1.400 (5)	C14—O19	1.385 (4)
C1—N9	1.406 (5)	C14—C15	1.387 (5)
C2—C3	1.371 (5)	C15—C16	1.394 (5)
C2—H2	0.93	C15—H15	0.93
C3—C4	1.365 (5)	C16—C17	1.377 (5)
C3—F7	1.320 (5)	C16—H16	0.93
C4—F8	1.361 (4)	C17—O21	1.386 (5)
C4—C5	1.369 (5)	C17—C18	1.391 (5)
C5—C6	1.369 (5)	C18—H18	0.93
C5—H5	0.93	O19—C20	1.434 (5)
C6—H6	0.93	C20—H20A	0.96
N9—C10	1.362 (4)	C20—H20B	0.96
N9—H9	0.84 (4)	C20—H20C	0.96
C10—O11	1.235 (4)	O21—C22	1.439 (4)
C10—N12	1.370 (5)	C22—H22A	0.96
N12—C13	1.418 (5)	C22—H22B	0.96
N12—H12	0.80 (4)	C22—H22C	0.96
C13—C18	1.385 (5)		
			
C2—C1—C6	119.3 (4)	C14—C13—N12	117.5 (4)
C2—C1—N9	123.2 (3)	O19—C14—C15	125.3 (4)
C6—C1—N9	117.5 (3)	O19—C14—C13	114.7 (4)
C3—C2—C1	119.0 (3)	C15—C14—C13	120.1 (4)
C3—C2—H2	120.5	C14—C15—C16	120.0 (4)
C1—C2—H2	120.5	C14—C15—H15	120
F7—C3—C4	118.0 (4)	C16—C15—H15	120
F7—C3—C2	120.9 (4)	C17—C16—C15	119.7 (4)
C4—C3—C2	121.1 (4)	C17—C16—H16	120.2
C4—C3—H3	119.4	C15—C16—H16	120.2
C2—C3—H3	119.4	C16—C17—O21	124.7 (4)
F8—C4—C3	119.4 (4)	C16—C17—C18	120.7 (4)
F8—C4—C5	120.1 (4)	O21—C17—C18	114.6 (4)
C3—C4—C5	120.5 (4)	C13—C18—C17	119.7 (4)
C6—C5—C4	119.9 (4)	C13—C18—H18	120.1
C6—C5—H5	120	C17—C18—H18	120.1
C4—C5—H5	120	C14—O19—C20	116.7 (3)
C5—C6—C1	120.1 (4)	O19—C20—H20A	109.5
C5—C6—H6	119.9	O19—C20—H20B	109.5
C1—C6—H6	119.9	H20A—C20—H20B	109.5
C10—N9—C1	126.7 (3)	O19—C20—H20C	109.5
C10—N9—H9	115 (3)	H20A—C20—H20C	109.5
C1—N9—H9	118 (3)	H20B—C20—H20C	109.5
O11—C10—N9	123.7 (4)	C17—O21—C22	115.9 (3)
O11—C10—N12	122.9 (4)	O21—C22—H22A	109.5
N9—C10—N12	113.4 (3)	O21—C22—H22B	109.5
C10—N12—C13	125.9 (3)	H22A—C22—H22B	109.5
C10—N12—H12	120 (3)	O21—C22—H22C	109.5
C13—N12—H12	114 (3)	H22A—C22—H22C	109.5
C18—C13—C14	119.9 (4)	H22B—C22—H22C	109.5
C18—C13—N12	122.6 (4)		

### Hydrogen-bond geometry (Å, °)

**Table:**

*D*—H···*A*	*D*—H	H···*A*	*D*···*A*	*D*—H···*A*
N9—H9···O11^i^	0.84 (4)	2.09 (4)	2.907 (4)	163 (4)
N12—H12···O11^i^	0.80 (4)	2.30 (4)	3.002 (4)	147 (4)

Symmetry codes: (i) *x*, −*y*+1/2, *z*+1/2.
